# Corrosion and Wear Behavior of WC–10Co4Cr Coating under Saturated Salt Drilling Fluid

**DOI:** 10.3390/ma14237379

**Published:** 2021-12-02

**Authors:** Hao Yin, Jian Liang, Xiaoyong Ren, Jie Zhao, Xin He, Yanhong Gu

**Affiliations:** 1 Institute of Exploration Techniques, Chinese Academy of Geological Sciences, Langfang 065000, China; liangjian1@mail.cgs.gov.cn; 2 School of Mechanical Electronic & Information Engineering, China University of Mining & Technology (Beijing), Beijing 100083, China; 3 School of Mechanical Engineering, Beijing Institute of Petrochemical Technology, Beijing 102617, China; zhaojie@bipt.edu.cn (J.Z.); hexin02@spic.com.cn (X.H.); gu_yanhong@hotmail.com (Y.G.)

**Keywords:** corrosion resistance, erosion resistance, wear resistance, coating, drill pipe

## Abstract

Coating on the surface is one of the main ways to improve the corrosion resistance and wear resistance of materials. In this work, the corrosion, erosion, and wear resistance of WC–10Co4Cr coating and 27CrMoV substrate were compared by simulating the actual working conditions of the drill pipe. The simulation results show that the most serious corrosion occurred at the pipe body and the dominating erosion arose at the pipe joint closing to the inlet of the flow field. WC–10Co4Cr coating has excellent protection to 27CrMoV substrate, resulting in a 400 mV increase in corrosion potential, a two-orders-of-magnitude decrease in the corrosion current, and four times the improvement of the impedance value. The erosion resistance of the WC–10Co4Cr coating increased to more than 30% higher than that of the 27CrMoV substrate. The friction coefficient of the WC–10Co4Cr coating was much lower than that of the 27CrMoV substrate, and the wear resistance of the coating was higher than that of the substrate.

## 1. Introduction

Drill pipes are components used to connect the rig’s surface equipment to the drill bit underground [1,2,3]. Generally, they not only have to transfer huge bit weights and withstand great torsional stress, but they also suffer from erosion, corrosion, and wear during the work process [4,5,6]. Drilling fluid could cause the significant erosion and corrosion of a drill pipe, especially saturated saltwater, which is usually applied as drilling fluid in permafrost and salt rock drilling [1,7,8,9,10,11]. Moreover, the diameter of the pipe joint is usually larger than that of the drill pipe, resulting in significant wear. Hence, it is very urgent to enhance the erosion, corrosion, and wear resistance of the drill pipe [12]. 

Coating on the surface with excellent wear resistance and good protective effects is considered one of the most effective methods to improve the service performance of components in fields such as agricultural machinery, energy, transportation, chemical industry, and drilling engineering. Hence, a hard coating could be applied to the surface of the drill pipe or joint to improve its anti-corrosion and anti-wear [8,13,14,15,16]. There are several major coating methods to prepare hard coatings, such as high-velocity oxygen–fuel spraying [1,17], cold spraying [15,18], plasma spraying [19], micro-arc oxidation [20,21], high-speed laser cladding [22], high-power impulse magnetron sputtering [16], plasma electrolytic oxidation [23], and so on [13,24]. WC-based cemented carbide possesses high hardness, outstanding strength, and excellent toughness, so is widely used as anti-wear material [1,8,17]. WC-based coatings are often coated on the surface of the pipe to increase its wear and corrosion resistance. 

The working conditions of the drill pipe are very complicated. Erosion, electrochemical corrosion, and wear of the surface by the drilling fluid may occur at the same time during operation [25,26,27]. Although many studies have been carried out on the properties of WC-based coatings, there is still a lack of systematic evaluation of the erosion, corrosion, and wear resistance of WC-based coatings under working conditions. In addition, the investigation by a combination of experiment and simulation on the corrosion and erosion distribution and rate is still scarce, but is very meaningful. 

In the present study, the corrosion, erosion, and wear behaviors of 27CrMoV steel substrate and WC–10Co4Cr coating have been studied using experiment and simulation methods. A saturated saltwater solution was used as a simulation drilling fluid. The erosion and corrosion rates were evaluated to guide the application of WC–10Co4Cr coating in actual working conditions.

## 2. Materials and Methods

### 2.1. Simulation

**Figure 1:**
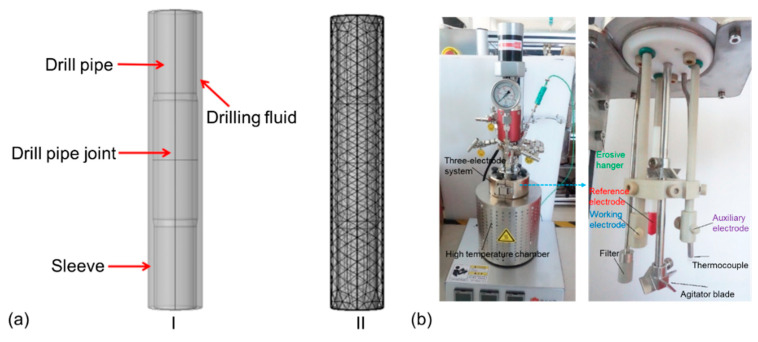
(**a**) The constructed model and meshing strategies for computational domain of drill pipe. (**b**) The test device of the erosion and corrosion resistance in this work.

The corrosion and erosion behavior of drill pipe was simulated with Comsol Multiphysics 5.5 finite element software [28]. Considering the influence of the electrochemical corrosion, flow erosion, and temperature field, the corrosion and erosion behavior of drill pipe under long periods of operation is predicted. The constructed model is shown in Figure 1a, including the drill pipe, drill pipe joint, sleeve, and drilling fluid. The user-defined control grid method was used to divide the grid. The boundary layer number, the tension factor, and the thickness adjustment factor were 6, 1.2, and 2, respectively. As the corrosion of the outer and outer pipe walls is serious and harmful in real working conditions, the inner wall corrosion was ignored in this simulation. 

### 2.2. Corrosion and Erosion Characterization 

In this work, 27CrMoV alloy steel was used as the substrate. Table 1 shows the chemical composition of 27CrMoV. The WC–10Co4Cr coating was sprayed on the substrate by high-velocity oxygen–fuel (HVOF) spraying method (GTV Impex GmbH, Germany). Commercial WC–10Co4Cr powder (86 wt.% WC, 10 wt.% Co, and 4 wt.% Cr) was used as the spraying powder (produced by BGRIMM Technology Group, Beijing, China). Before spraying, the substrate was ultrasonically cleaned in acetone. The specific spraying parameters were: spraying distance 420 mm, powder feed rate 90 g/min, oxygen flow rate 900 L/min, and kerosene flow rate 26 L/min. 

Saturated saline solution with pH 10 was used as the drilling fluid, which consisted of saturated NaCl, 2 wt.% salt-resistant bentonite, 2 wt.% filtrate reducer, and a certain amount of NaOH. The electrochemical measurements were carried out in a three-electrode cell (AMETEK, San Diego, California, U.S.), and the test device is shown in Figure 1b. A total of 200 mL saturated saline solution was added into the high-temperature chamber. A platinum sheet and saturated calomel electrode were used as counter electrode and reference electrode, respectively. The samples were installed onto the working electrode and erosive hanger. Then, the chamber was sealed off. The experimental temperature was set at 120 °C, and the speed of the agitator blade was set at 250 rpm. The open-circuit potentials (OCP) test was conducted for 30 min, and then electrochemical impendence spectroscopy (EIS) was measured. The frequency range was set to 100 kHz to 10 MHz, and the perturbation amplitude of the additional sine wave was 10 mV. Dynamic potential polarization test was conducted with a potential scan rate of 0.5 mV/s and range from −250 mV to 500 mV. The test was carried out for 7 days without halt. The impedance measured was fitted by the software of VERSASTAT3F workstation, and the polarization curve data were fitted by Tafel method. Erosion test was carried out together with the corrosion test. The weight of the samples was measured before and after erosion test to evaluate the erosion resistance of the sample. Three groups of the samples were tested to ensure the reproducibility of the corrosion and erosion test. 

### 2.3. Friction Test

The MS-T3001 multi-functional tribometer (Lanzhou Huahui Instrument Technology Co., Ltd, Lanzhou, China) was used to study the wear behavior of the 27CrMoV substrate and WC–10Co4Cr coating. The SiC ball of 2 mm diameter was used as the counterpart. The test load was 1000, 1500, and 2000 g. The rotation mode with a speed of 120 rpm and rotation radius of 2 mm was used in the test. The experiment was carried out three times under the same parameters to eliminate the influence of human error. Three-dimensional morphology of the wear surface was observed to calculate the wear volume and analyze the wear mechanism.

## 3. Results

### 3.1. Corrosion and Erosion Behavior

**Figure 2:**
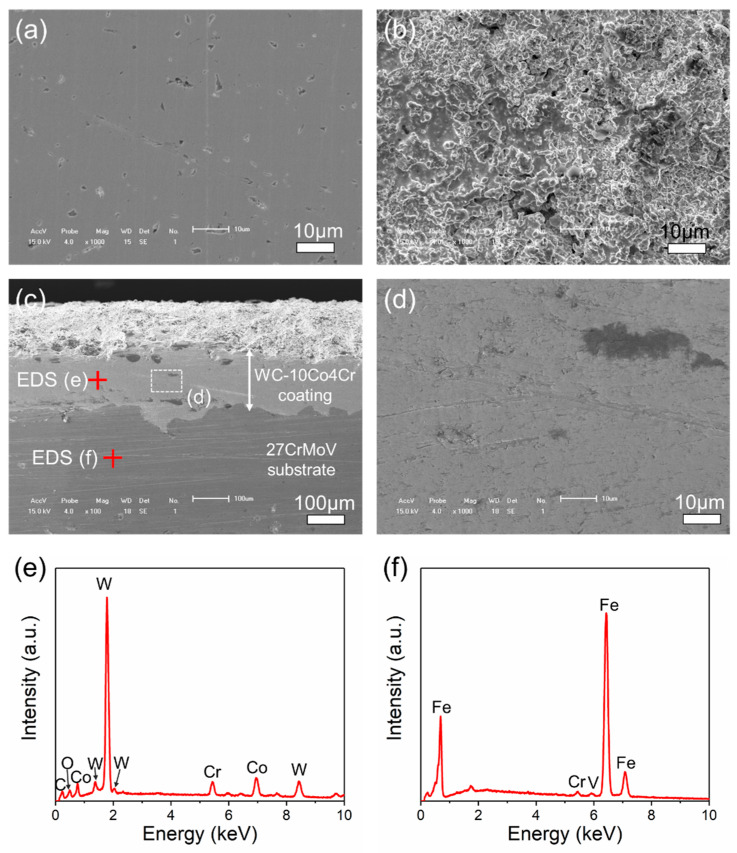
Typical SEM images of surface micro-morphology of (**a**) 27CrMoV substrate and (**b**) WC–10Co4Cr coating. (**c**) SEM images of the cross-section of the WC–10Co4Cr coating and (**d**) the magnified view of the part as marked in (**c**). (**e**,**f**) EDS patterns of the coating and substrate as marked in (**c**).

Figure 2a,b shows the surface micro-morphology of the 27CrMoV substrate and the obtained WC–10Co4Cr coating. The cross-section of the WC–10Co4Cr coating is presented in Figure 2c,d. It can be seen that the surface of the obtained coating was rough, with many particles and micropores on the surface. However, the coating was relatively dense inside, as shown in Figure 2d. The thickness of the coating was about 200 μm, and there were no obvious interface cracks between the coating and substrate. Figure 2e,f shows the EDS patterns of the coating and the substrate. The elements of W, Co, Cr, and C were detected in the coating, and the W shows the highest relative intensity. In the substrate, Fe was the main element, and Cr and Mo were also detected with weak intensity.

**Figure 3:**
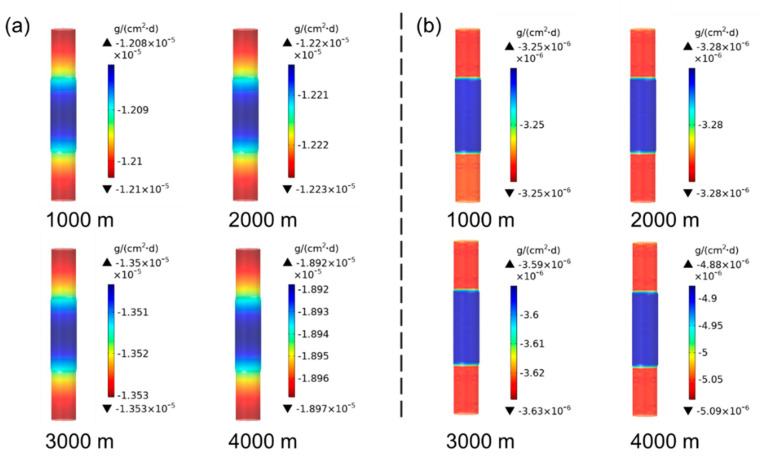
Corrosion rate distributions of the (**a**) 27CrMoV substrate and (**b**) WC–10Co4Cr coatings at different drilling depths for the drill pipe obtained by simulation.

Firstly, the corrosion statuses of the drill pipes were simulated, which stayed at a depth ranging from 1000 to 4000 m for 40 days. Normally, the drilling pipe temperature increases by 30 °C for each 1000 m of drilling depth. Therefore, the corrosion of the drill pipe at different depths corresponds to the influence of different temperatures on its corrosion. Figure 3 shows the simulated results of the corrosion rate distributions of the 27CrMoV substrate and WC–10Co4Cr coatings at different drilling depths. For the same depth, the drill pipe body corrodes more severely than the pipe joint. The reasons for the drill pipe corrosion mainly include galvanic corrosion between the sleeve and the drill pipe and the electrochemical corrosion in electrolytic solution [29]. The diameter of the drill pipe body is smaller than that of the joint, thus affecting galvanic corrosion. Compared with 27CrMoV substrate and WC–10Co4Cr coatings, the corrosion rate distributions are basically the same. 

**Figure 4:**
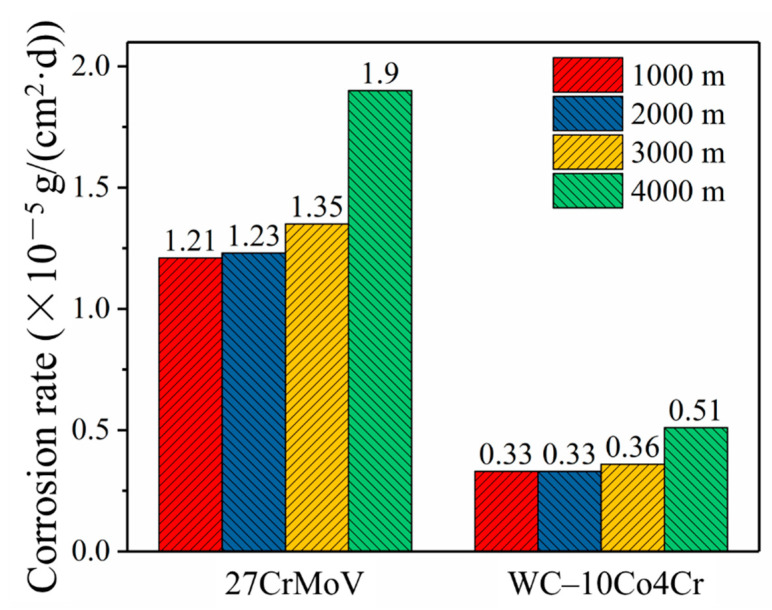
Comparison of the corrosion rates of 27CrMoV substrate and WC–10Co4Cr coatings at different drilling depths obtained by simulation.

Figure 4 shows the values of the corrosion rates of the 27CrMoV substrate and WC–10Co4Cr coatings obtained by simulation. It can be seen that the corrosion of the drill pipe gradually increased with the increase in drilling depth. However, the corrosion rate of the WC–10Co4Cr coatings was about one order of magnitude lower than that of the 27CrMoV substrate. The self-corrosion potential of the WC–10Co4Cr coatings was higher than that of the sleeve material. It was the cathode in galvanic corrosion, resulting in negligible galvanic corrosion. The main reason for corrosion was the electrochemical corrosion in the drilling fluid.

**Figure 5:**
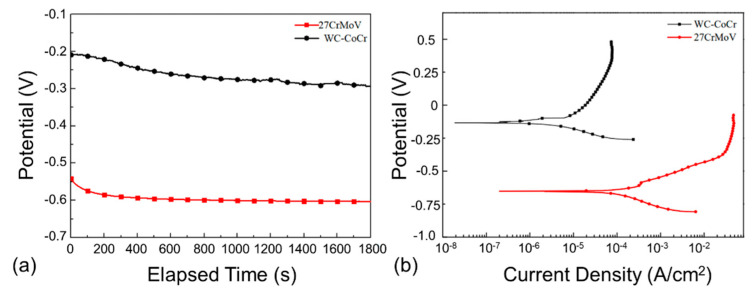
(**a**) Time dependence of open-circuit potentials and (**b**) potentiodynamic polarization curves of the 27CrMoV substrate and WC–10Co4Cr coatings measured in saturated saline solution.

In order to analyze the corrosion behavior of the 27CrMoV substrate and WC–10Co4Cr coating, the electrochemical behavior was tested at 120 °C by experiment. Figure 5a shows the experimental results of the open-circuit potentials with the test in saturated saline solution. It is clear that the open-circuit potential of the WC–10Co4Cr coating was much higher than that of the 27CrMoV substrate, indicating that the corrosion tendency of the coating was less than that of the 27CrMoV substrate. The WC–10Co4Cr coating possessed better anti-corrosion performance than that of the 27CrMoV substrate. 

Figure 5b shows the potentiodynamic polarization (PDP) curves of the 27CrMoV substrate and WC–10Co4Cr coatings measured in a saturated saline solution. Both of the samples were in active states and did not exhibit the typical passive behavior. The corrosion potential of the WC–10Co4Cr coatings was about −140 mV, which is about 521 mV higher than that of the 27CrMoV substrate (about −661 mV). The corrosion current densities are 2.8 and 107.1 μA/cm^2^ for the WC–10Co4Cr coating and 27CrMoV substrate, respectively. The corrosion current density of WC–10Co4Cr coating decreased by two orders of magnitude compared to that of the 27CrMoV substrate, resulting in a significant decrease in the corrosion rate of the WC–10Co4Cr coating. Therefore, the WC–10Co4Cr coating has better corrosion resistance than the 27CrMoV substrate. In corrosive working conditions, the drill pipe with WC–10Co4Cr coating will have a longer service life than that without the coating. 

**Figure 6:**
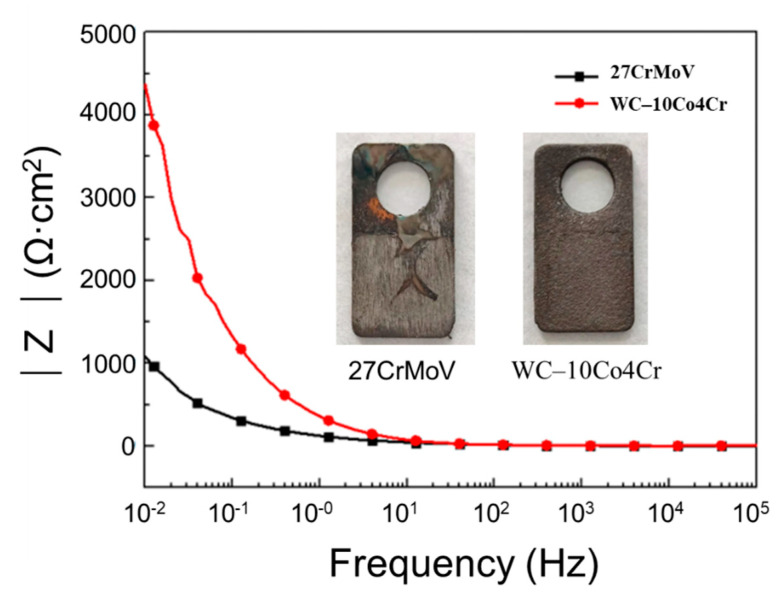
Bode diagram of EIS of 27CrMoV and WC–10Co4Cr coatings measured in saturated saline solution. The inset images show the macroscopic morphology of the sample after the corrosion test.

Figure 6 displays the experimental result of the Bode diagrams derived from electrochemical impedance spectroscopy. The values of impedance magnitude of the 27CrMoV substrate and the WC–10Co4Cr coating are almost the same at high frequency. However, at low frequency, the impedance of the WC–10Co4Cr coating is 3000 Ω·cm^2^ lower than that of the 27CrMoV substrate. This also indicates that the WC–10Co4Cr coating has better corrosion resistance than the 27CrMoV substrate. The inset images in Figure 6 show the macroscopic morphology of the 27CrMoV substrate and the WC–10Co4Cr coating after the corrosion test. The WC–10Co4Cr coating shows light corrosion marks, and some scour marks can be seen on the 27CrMoV substrate surface.

**Figure 7:**
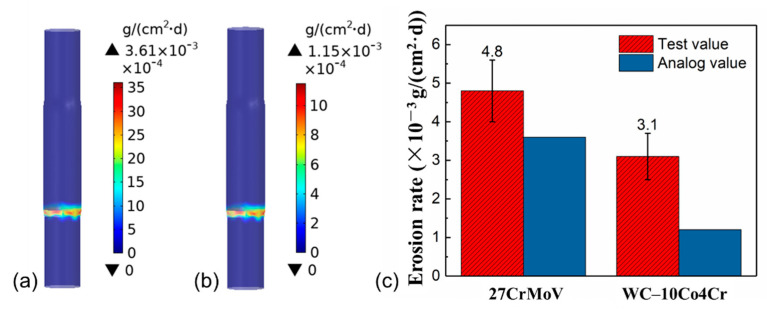
Erosion rate distributions of the (**a**) 27CrMoV substrate and (**b**) WC–10Co4Cr coatings for the drill pipe obtained by simulation. (**c**) Comparison of the erosion rates of 27CrMoV substrate and WC–10Co4Cr coatings obtained by simulation and experiment.

The erosion behavior of the drill pipe under drilling fluid conditions was analyzed by simulation and experiment. Figure 7a,b shows the simulated results of the erosion rate distributions of 27CrMoV and WC–10Co4Cr coating for the drill pipe. It can be seen that the most serious erosion occurred in the pipe joint area, which is close to the inlet of the flow field and with large geometric deformation. This area is subject to maximum fluid scour from the drill fluid. Figure 7c gives the values of the erosion rate obtained by simulation (analog value) and experiment (test value). The test values of the erosion rate are 4.8 and 3.1 mg/(cm^2^·d) for 27CrMoV and WC–10Co4Cr coating, respectively. The analog values of the erosion rate are 3.6 and 1.2 mg/(cm^2^·d) for 27CrMoV and WC–10Co4Cr coating, respectively. The difference between the test and analog values is due to the measurement error and the setting of boundary conditions. However, both the results show that the erosion resistance of the coating is better than that of the substrate. The erosion resistance of the coating increased to more than 30% higher than that of the 27CrMoV substrate. 

### 3.2. Tribological Performance

**Figure 8:**
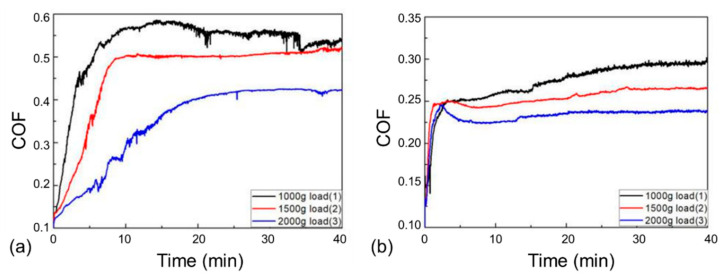
Friction coefficients of the (**a**) 27CrMoV substrate and (**b**) WC–10Co4Cr coatings at different loads.

**Figure 9:**
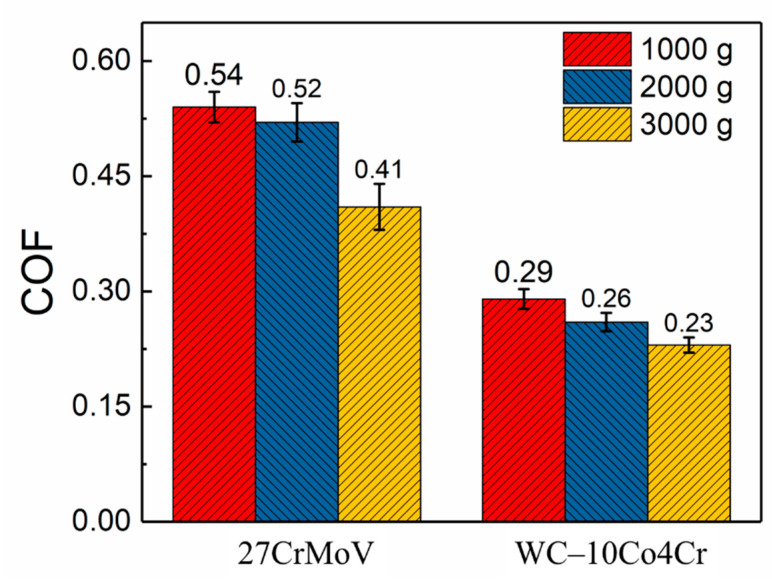
The average coefficient of friction after the running-in stage of the 27CrMoV substrate and WC–10Co4Cr coatings at different loads.

Figure 8 presents the friction coefficient curves of the 27CrMoV substrate and WC–10Co4Cr coating at different loads. There is a stage of a rapid increase in friction coefficients at the beginning of the friction test, usually called the running-in stage. The friction coefficients of both the 27CrMoV substrate and the WC–10Co4Cr coating increased sharply in the running-in stage and then remained stable. Figure 9 shows the average COF value of each sample after the running-in stage. It can be seen that the COFs of the 27CrMoV substrate were much higher than that of the WC–10Co4Cr coating at the corresponding load. The COF of the coating at 2000 g load was about 0.225, which is 0.18 lower than that of the substrate (about 0.4). Therefore, the WC–10Co4Cr coating has better wear-reduction performance than the substrate. 

**Figure 10:**
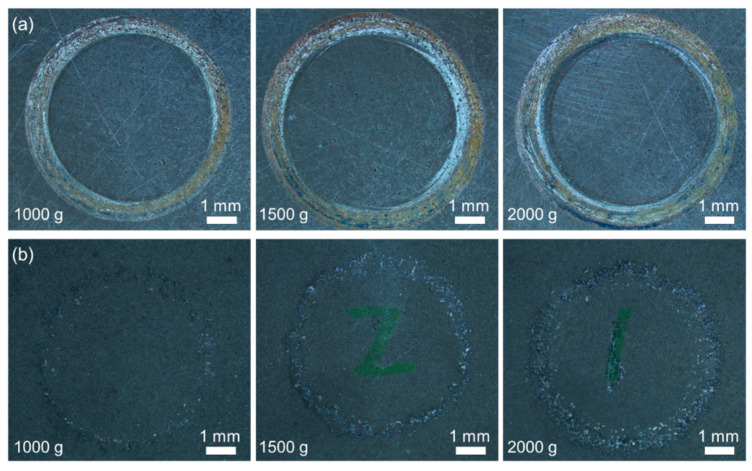
Surface morphologies of the (**a**) 27CrMoV substrate and (**b**) WC–10Co4Cr coatings after friction test at different loads.

Figure 10 shows the macroscale wear tracks on the 27CrMoV substrate and WC–10Co4Cr coating at different loads. With the increase in the applied load, the wear of the samples gradually became significant. On the whole, the wear of the coating was less than that of the substrate. According to the statistics, the widths of the wear track on the 27CrMoV substrate at 1000, 1500, and 2000 g loads were 821, 1184, and 1495 μm, respectively. The widths of the wear track on the WC–10Co4Cr coating at 1000, 1500, and 2000 g loads were 458, 568, and 935 μm, respectively. The WC–10Co4Cr coating exhibits better wear resistance than the 27CrMoV substrate.

**Figure 11:**
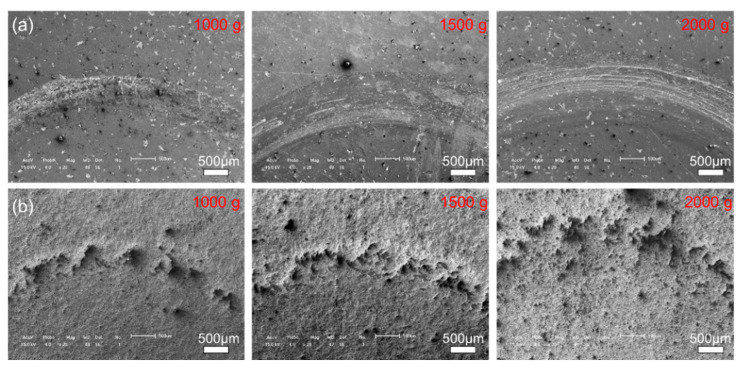
Typical SEM images of the wear track of the (**a**) 27CrMoV substrate and (**b**) WC–10Co4Cr coatings after friction test at different loads.

**Figure 12:**
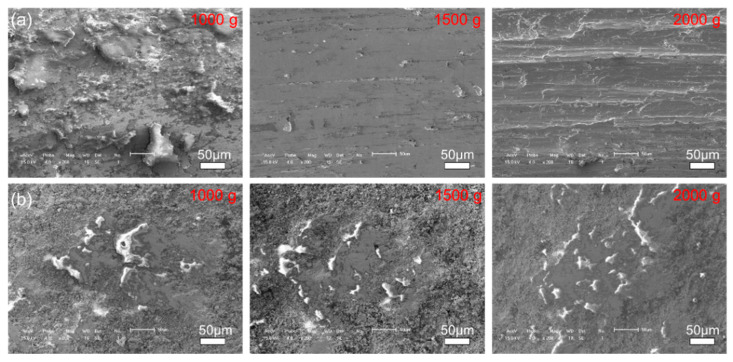
The morphologies of the wear surface of the (**a**) 27CrMoV substrate and (**b**) WC–10Co4Cr coatings after friction test at different loads.

**Figure 13:**
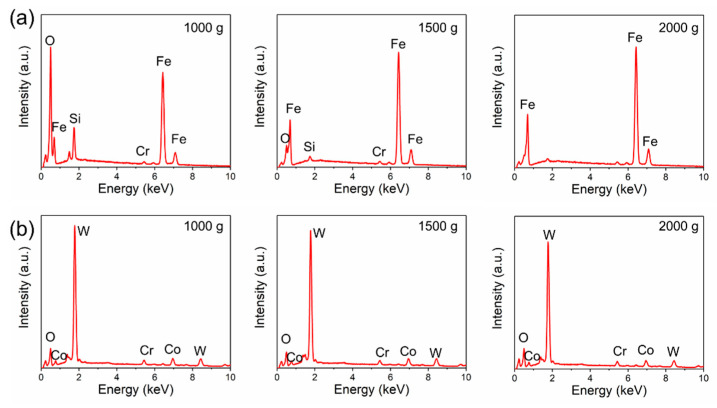
EDS patterns of the wear surface of the (**a**) 27CrMoV substrate and (**b**) WC–10Co4Cr coatings after friction test at different loads.

Figure 11 and Figure 12 show the morphologies of the wear tracks at higher multiples. It can be seen that the wear on the substrate is much more serious than that where the coating and the continuous wear track were formed. Grooves and scratches can be seen on the substrate. For the coating, several pits formed on the wear track, and the material transfer film formed partly, as shown in Figure 12. Figure 13 shows the EDS patterns of the wear surface of the substrate and coating after friction tests at different loads. The elements of Fe and Cr were detected on the wear surface of the substrate with a load of 1000 g. 

**Figure 14:**
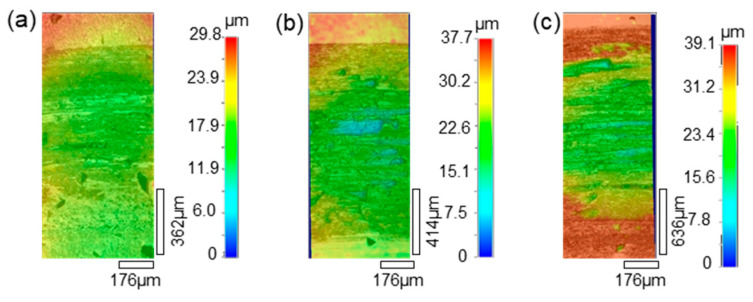
LSCM images of the wear tracks on the 27CrMoV substrate at (**a**) 1000 g, (**b**) 1500 g, and (**c**) 2000 g.

**Figure 15:**
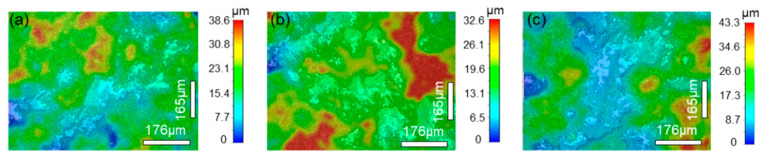
LSCM images of the wear tracks on the WC–10Co4Cr coating at (**a**) 1000 g, (**b**) 1500 g, and (**c**) 2000 g.

The morphologies of the wear tracks on the 27CrMoV substrate and WC–10Co4Cr coating are characterized by laser scanning confocal microscope (LSCM) and shown in Figure 14 and Figure 15. As shown in Figure 14, the wear tracks on the substrate are obvious and gradually deepen as the load increases. The scratch grooves on the wear track can be observed even at a 1000 g load. However, the wear tracks on the coating are shallow. Some scattered spalling pits can be seen on the wear tracks, and there is no obvious scratch groove. This also shows that the coating has better wear resistance than the substrate. 

**Figure 16:**
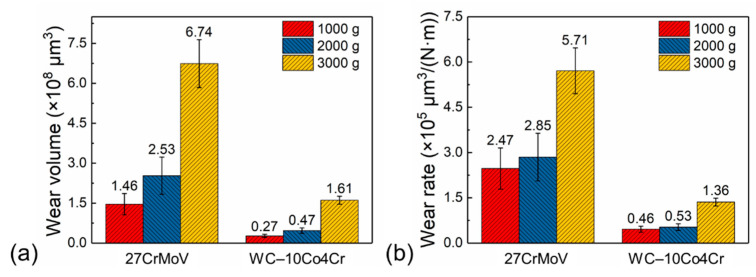
(**a**) Wear volumes and (**b**) wear rate of the 27CrMoV substrate and WC–10Co4Cr coating after friction tests at different loads.

The wear volumes and wear rate of the 27CrMoV substrate and WC–10Co4Cr coating after friction tests at various loads are shown in Figure 16. For the same sample, the wear volume increased gradually with the increase in load, which is consistent with the wear morphology. The wear volume decreased from 1.46 × 10^8^, 2.53 × 10^8^, and 6.74 × 10^8^ μm^3^ for the substrate to 0.27 × 10^8^, 0.47 × 10^8^, and 1.61 × 10^8^ μm^3^ for the coating at 1000, 1500, and 2000 g loads, respectively. The wear rate decreased from 2.47 × 10^5^, 2.85 × 10^5^, and 5.71 × 10^5^ μm^3^/(N·m) for the substrate to 0.46, 0.53, and 1.36 μm^3^/(N·m) for the coating at 1000, 1500, and 2000 g loads, respectively. The reduction in the wear volume and wear rate is attributed to the good lubrication effect and the excellent mechanical properties of the coating. 

## 4. Conclusions

The corrosion, erosion, and wear resistance of the WC–10Co4Cr coating and 27CrMoV substrate are studied in this work, and the following conclusions can be drawn:

(1) The WC–10Co4Cr coating shows great protection to 27CrMoV substrate. The corrosion potential and current of the coating increases by about 400 mV and decreases by two orders of magnitude compared with the 27CrMoV substrate;
(2) The erosion resistance of the coating increased to about 30% higher than that of the 27CrMoV substrate;
(3) The average friction coefficient of the 27CrMoV substrate is significantly higher than that of the WC–10Co4Cr coating. The wear resistance of the coating is much better than that of the substrate, and the wear volume is greatly reduced under the same friction conditions.

## Figures and Tables

**Table 1:** The chemical composition of the 27CrMoV substrate with the exception of the element of Fe.

Chemical Composition	C	Si	Mn	S	P	Cr	V	Mo
wt.%	0.23~0.31	0.2~0.4	0.3~0.6	≤0.04	≤0.04	1.2~1.5	0.15~0.3	0.5~0.6

## Data Availability

All data needed to evaluate the conclusions in the paper are present in the paper. Additional data related to this paper may be requested from the authors.
